# Transient Neurological Deficits in a Patient With Prior DeBakey Type 1 Aortic Dissection

**DOI:** 10.7759/cureus.67839

**Published:** 2024-08-26

**Authors:** Mehak Sharma, Adam Daren, Elizma Pretorius, Leila Keeler, Ilya Fonarov

**Affiliations:** 1 Internal Medicine, Florida International University, Herbert Wertheim College of Medicine, Miami, USA; 2 Internal Medicine, St. George's University, True Blue, GRD; 3 Obstetrics and Gynecology, Orlando College of Osteopathic Medicine, Winter Garden, USA; 4 Primary Care, Orlando College of Osteopathic Medicine, Orlando, USA; 5 Hospital Medicine, Jackson Memorial Hospital, Miami, USA

**Keywords:** emergency, dissection, aortic, stroke, debakey

## Abstract

Aortic dissection is a life-threatening emergency that occurs when the aortic wall layers are separated. It is important to recognize that aortic dissections can have an atypical presentation with neurological deficits and not typical findings of chest pain or thoracic symptoms. Our patient presented with headache, vertigo, and transient neurologic deficits. However, imaging revealed no acute stroke. Our patient had a prior history of DeBakey type 1 aortic dissection repaired four years before. Imaging did not demonstrate an acute aortic dissection however there was an increase in size of the aneurysmal components. However, it is unclear if this contributed to the transient neurological deficits. Further research is needed to determine if there is a correlation between aortic aneurysmal enlargement and transient neurological deficits.

## Introduction

A DeBakey type 1 aortic dissection begins in the ascending aorta, propagating to at least the aortic arch [1]. Aortic dissection has an incidence rate of 11.43 per one million annually [2]. Classically, aortic dissection is suspected when patients complain of severe or tearing chest pain that reaches its maximal intensity very quickly. However, despite being previously established as the most common symptom, approximately 6% of all cases with type A aortic dissection report no thoracic pain [3]. Early treatment is crucial since pain-free dissection is often diagnosed late [4]. Patients can also present with neurological symptoms and manifestations as an initial presentation, as highlighted in this case.

## Case presentation

A 54-year-old female presented to the emergency department with a severe thunderclap-like headache and vomiting lasting for one hour. She had a history of a DeBakey type 1 aortic dissection that was repaired with a bioprosthetic aortic valve replacement extending from the annulus of the aortic valve to the bilateral common iliac artery four years prior. Additionally, she has a history of hypertension, previous right frontal stroke, hypothyroidism, asthma, and a carotid aneurysm repaired 30 years ago. She denied the use of alcohol, tobacco, or other social drugs. Her home medications were carvedilol, losartan, aspirin, and rosuvastatin. Before the development of the headache, she became vertiginous with a questionable loss of consciousness. In addition, she reported an inability to move her legs and left arm. She denied chest pain or shortness of breath.

On the physical examination, her vital signs were a temperature of 37.9°C, heart rate of 67 beats/minute, blood pressure of 157/88 mmHg, and 100% oxygen saturation on room air. The patient was mildly distressed with slight left facial nasolabial fold flattening and increased muscle tone bilaterally, greater on the left than the right. Her strength was 5+/5 in the right upper and lower extremities and 4+/5 in the left upper and lower extremities. Her reflexes were slightly more brisk at the left bicep, left tricep, and left brachioradialis when compared to the right. The pulses were symmetrical. Otherwise, her cardiovascular, abdominal, and lung examinations were unremarkable.

**Figure 1:**
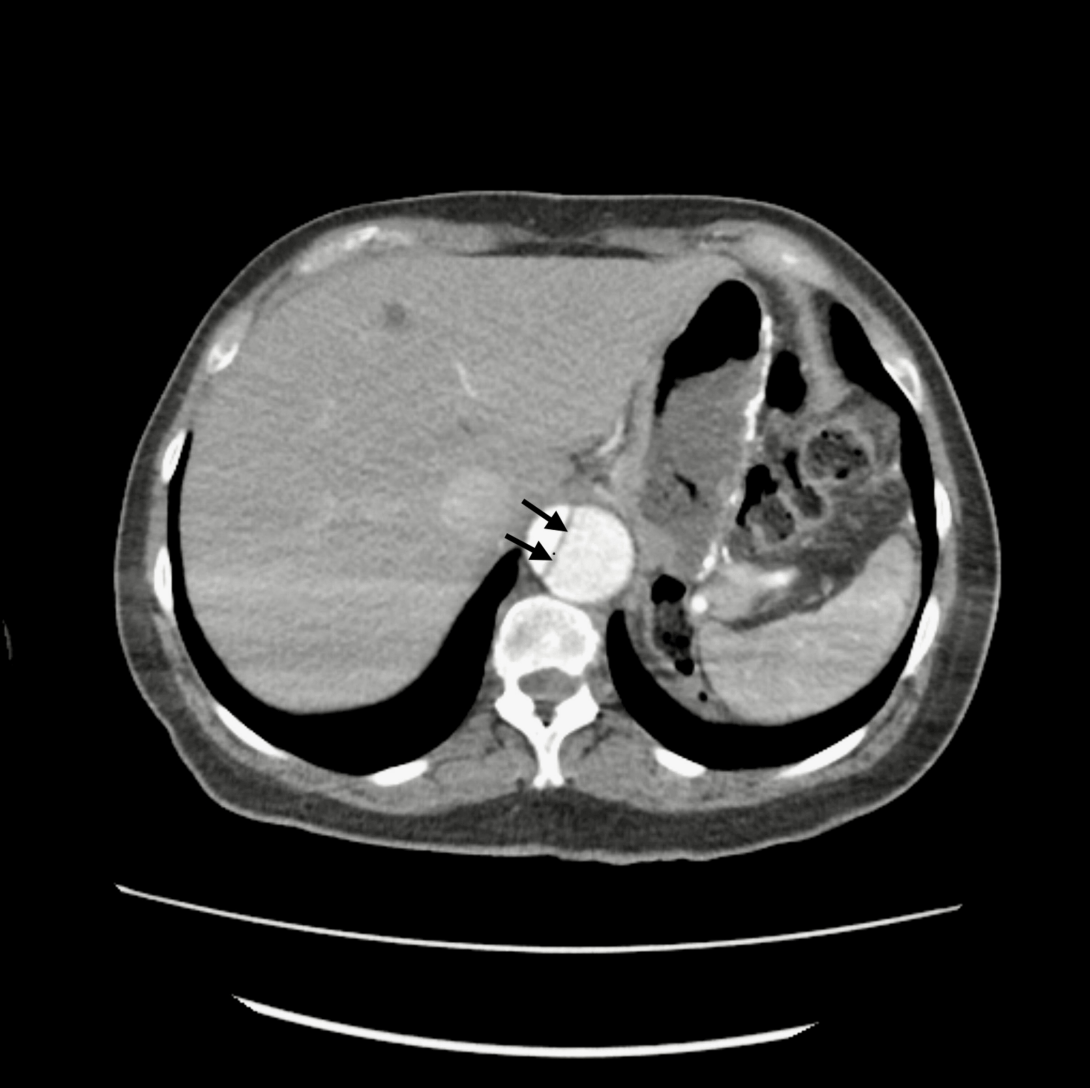
CTA scan axial images of the upper abdomen with a repaired DeBakey type 1 dissection. Arrows indicate the false lumen of the dissection CTA: computed tomography angiography

**Figure 2:**
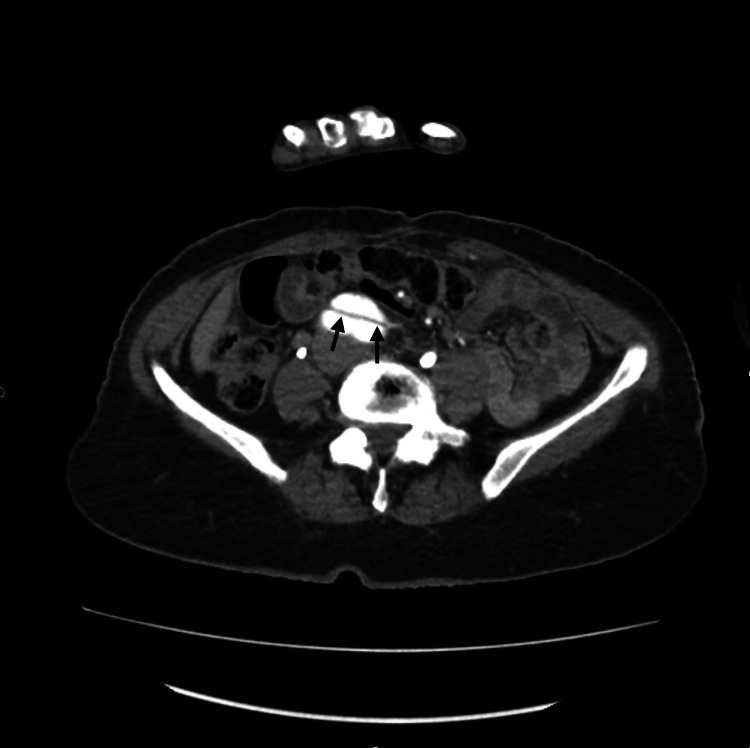
CTA axial image of the middle abdomen with a repaired DeBakey type 1 dissection. Arrows indicate the false lumen of the dissection CTA: computed tomography angiography

**Figure 3:**
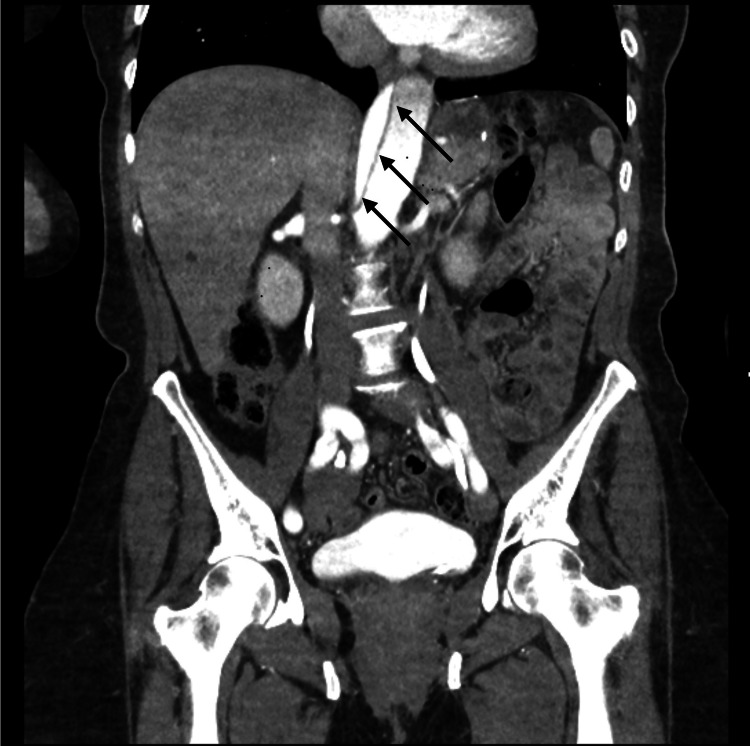
CTA of the abdomen and pelvis coronal image showing an alternative view of the repaired DeBakey type 1 dissection. Arrows indicate the false lumen of the dissection CTA: computed tomography angiography

**Figure 4:**
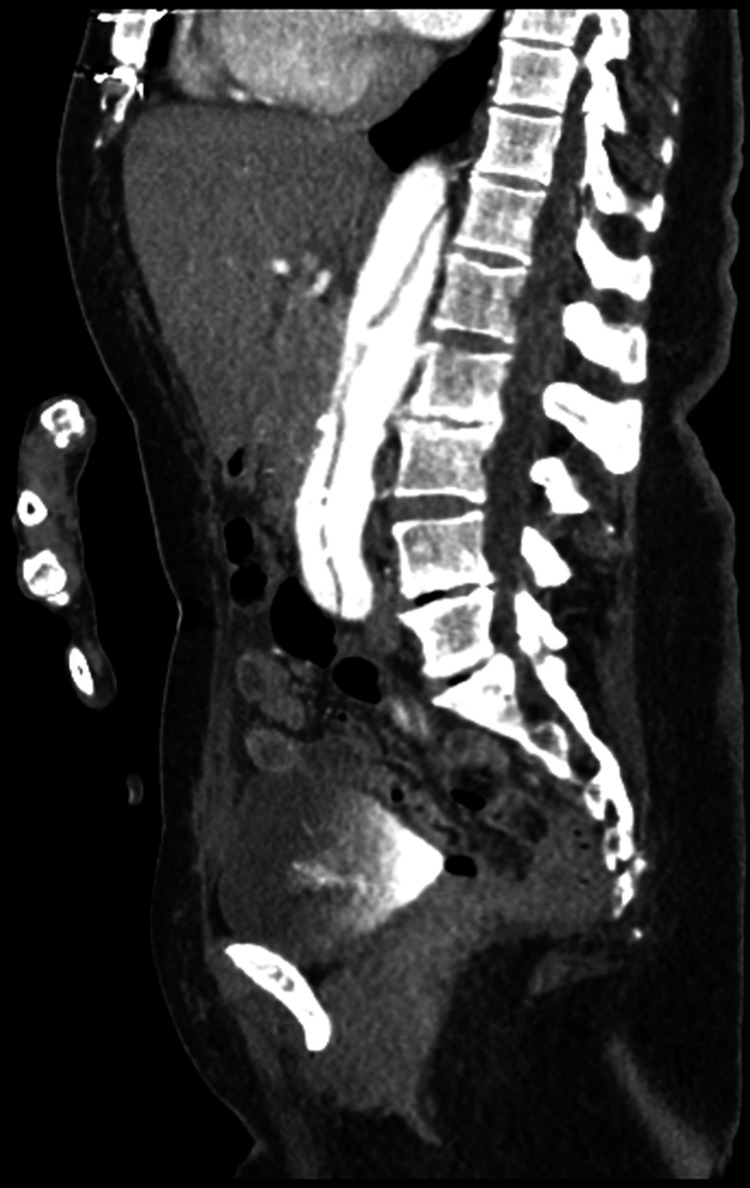
CTA of the abdomen and pelvis sagittal image with a repaired DeBakey type 1 dissection CTA: computed tomography angiography

The laboratory investigations were within the normal range except for the hemoglobin, which was slightly decreased (Table 1). The computed tomography (CT) scan of the brain without contrast demonstrated an old right frontal infarct without evidence of acute lesions. The magnetic resonance imaging of the brain without contrast was negative for acute territorial infarct, acute intracranial hemorrhage, or space-occupying lesion. The computed tomography angiography of the chest, abdomen, and pelvis revealed a preexisting repaired DeBakey type 1 dissection with evidence of underlying flow into the false lumen with increasing size of the aorta, especially the upper portion of the descending thoracic aorta, measuring 5.4 cm in diameter. The preexisting repaired DeBakey type 1 dissection of the thoracic and abdominal aorta was noted to have an increasing aneurysmal size of the descending thoracic component with a slight increase in the size of the distal abdominal aorta and common iliac arteries bilaterally. Figure 1 shows the upper abdomen axial view of the repaired aortic dissection. Figure 2 shows the middle abdomen axial view of the repaired aortic dissection. Figure 3 shows the abdominal coronal view of the repaired aortic dissection. Figure 4 shows the abdominal sagittal view of the repaired aortic dissection.

**Table 1:** Relevant laboratory results upon investigation PT: prothrombin time; INR: international normalized ratio; PTT: partial thromboplastin time; WBC: white blood cell count; ESR: erythrocyte sedimentation rate

Test	Patient’s values	Normal range
Troponin	<0.012 ng/mL	0-0.034 ng/mL
PT	14.7 seconds	10-13 seconds
INR	1.13	0.8-1.1
PTT	29 seconds	25-35 seconds
WBC	11.4 x 10^3^/mcL	4.5-11 x 10^3^/mcL
Hemoglobin	11.6 g/dL	12-16 g/dL
ESR	18 mm/hour	0-20 mm/hour
Urine toxicology	Negative	Negative

Following these results, the patient was admitted to the medical intensive care unit in the setting of an abdominal aorta with increasing aneurysmal size. The patient was placed on esmolol and nicardipine infusions and then started on oral candesartan and carvedilol. The patient’s neurologic symptoms resolved, and an infectious workup was negative. The patient was evaluated by cardiothoracic surgery, and they determined that she was not a candidate for surgical intervention despite the change in the size of the aneurysmal component.

The patient denied any new or ongoing neurological symptoms since admission to the hospital, and she was discharged in a stable condition on her home medications. The patient was advised to follow up as an outpatient with her cardiologist and have repeat CT imaging in six months.

## Discussion

Our patient presented with thunderclap headache, vertigo, and neurologic deficits. Laboratory investigations were unrevealing; in this case, the patient had normal labs except for a slightly decreased hemoglobin level. The imaging was negative for an acute stroke. Due to the patient’s prior history of DeBakey type 1 aortic dissection that was repaired, the patient underwent imaging to rule out an acute dissection. Imaging ruled out an acute dissection of the thoracic and abdominal aorta. It is important to recognize that aortic dissections can have an atypical presentation with neurologic deficits and not typical findings of chest pain or thoracic symptoms. Patients with aortic dissection can present with neurologic symptoms at onset (17%-40% of patients) [4,5]. Neurologic deficits are present in about one-fifth of patients and typically include syncope and altered mental status [1]. In one study, neurologic symptoms and the initial manifestation in patients with type A aortic dissection included ischemic stroke, spinal cord ischemia, ischemic neuropathy, and hypoxic encephalopathy. [4].

A few case reports highlight the atypical presentation of aortic dissection, which presents with neurologic deficits. One case report is of a middle-aged male who had presented with altered consciousness and seizures. The patient was found to have an aortic dissection extending to the carotids [6]. Another report was a 65-year-old woman who reported left-sided weakness and was found to have a stroke in the right carotid artery. Her imaging also revealed an aortic dissection extending to the right carotid artery [7]. A third case reported a 61-year-old woman who presented with a facial droop, left-sided weakness, and no thoracic pain. The patient was found to have aortic dissection, and the patient’s neurologic symptoms were attributed to the dissection [8].

Patients with aortic dissection are at risk for stroke in the immediate postoperative period. In the analysis by Ghoreishi et al., there was a 13% incidence of postoperative stroke. A longer period of circulatory arrest, cerebral perfusion, and cardiopulmonary bypass were all associated with an increased risk of postoperative stroke [9]. The long-term risk of stroke in these patients was investigated in the analysis by Chiappini et al. During the 27-year interval, out of the 380 patients that survived the initial surgical repair for an acute type A aortic dissection, 12 patients died of stroke [10].

Our patient was noted to have an increasing aneurysmal size of the descending thoracic component. In addition, there was a slight increase in the size of the distal abdominal aorta and common iliac arteries bilaterally on imaging. In the analysis by Halstead et al., of the 179 patients with a type A dissection repair studied over 17 years, 16 patients required reoperation due to aneurysm size or patient symptoms. The median diameters are slightly larger after the type A dissection repair, with an average growth rate of about 1 mm per year [11].

In the analysis by Fattori et al., the annual increase in aortic size was highest in the descending aortic section (0.37 ± 0.43 cm). It was notably greater when there was no blood clot in the false lumen (0.56 ± 0.57 cm) [12], thus supporting the need for continued surveillance of aneurysmal dilations with higher risk features. However, it is unclear if the aneurysmal changes contributed to the transient neurological deficits that the patient experienced. Further research is needed to determine if there is a correlation between aortic aneurysmal enlargement and transient neurological deficits.

## Conclusions

This case highlights the importance of imaging in evaluating changes in aortic aneurysmal diameter and the risks of aortic dissection. Although most patients report chest pain with acute aortic dissection, there are also more atypical presentations of acute aortic dissection, such as those with neurological symptoms where there is no report of thoracic pain. The presence of neurological symptoms may mask an acute aortic dissection and complicate the overall clinical picture. It is crucial to consider aortic dissection in patients who present with a prior history of aortic dissection. Further investigation is needed to determine if there is a correlation between aortic aneurysmal enlargement and transient neurological deficits. Patients with aneurysmal dilations that have higher risk features would benefit from close follow-up and imaging surveillance.
